# Supplementary material for: TMEM176B Regulates AKT/mTOR Signaling and Tumor Growth in Triple-Negative Breast Cancer
Source: Cells. 2021 Dec 6;10(12):3430. doi: 10.3390/cells10123430 (PMC8700203; doi:10.3390/cells10123430)
Supplement: Supplementary file 1 [file cells-10-03430-s001.zip › cells-1442375-supplementary.pdf]

Figure S1.

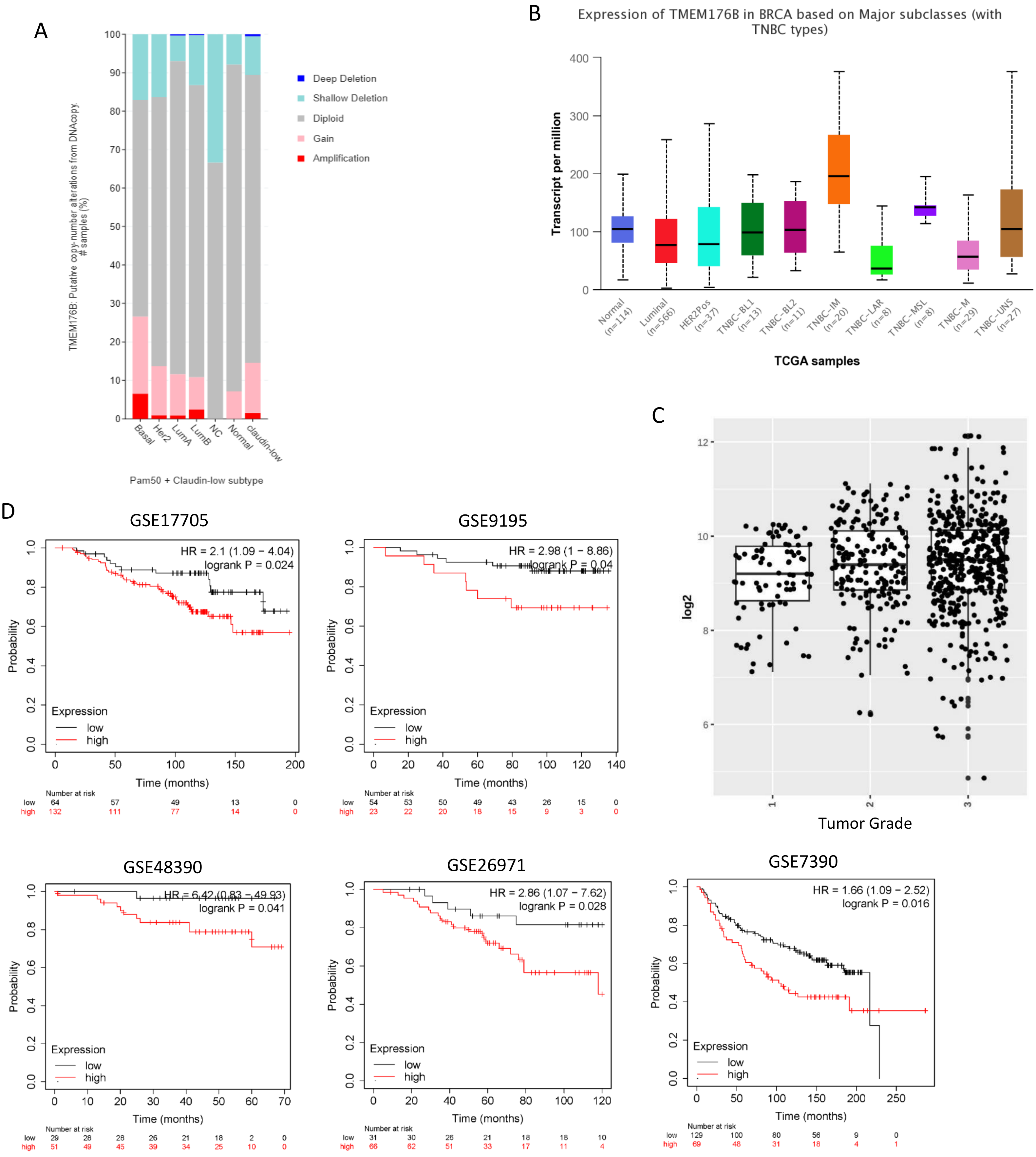
A
TMEM176B: Putative copy-number alterations from DNAcopy. # samples (%)
Deep Deletion
Shallow Deletion
Diploid
Gain
Amplification
Basal
Her2
LumA
LumB
NC
Normal
claudin-low
Pam50 + Claudin-low subtype
B
Expression of TMEM176B in BRCA based on Major subclasses (with TNBC types)
Transcript per million
Normal (n=114)
Luminal (n=566)
HER2Pos (n=37)
TNBC-BL1 (n=13)
TNBC-BL2 (n=11)
TNBC-IM (n=20)
TNBC-LAR (n=8)
TNBC-MSL (n=8)
TNBC-M (n=29)
TNBC-UNS (n=27)
TCGA samples
C
log2
Tumor Grade
D
GSE17705
HR = 2.1 (1.09 − 4.04)
logrank P = 0.024
GSE9195
HR = 2.98 (1 − 8.86)
logrank P = 0.04
GSE48390
HR = 6.42 (0.83 − 49.93)
logrank P = 0.041
GSE26971
HR = 2.86 (1.07 − 7.62)
logrank P = 0.028
GSE7390
HR = 1.66 (1.09 − 2.52)
logrank P = 0.016
Probability
Expression
low
high
Time (months)
Number at risk

Figure S1.

E

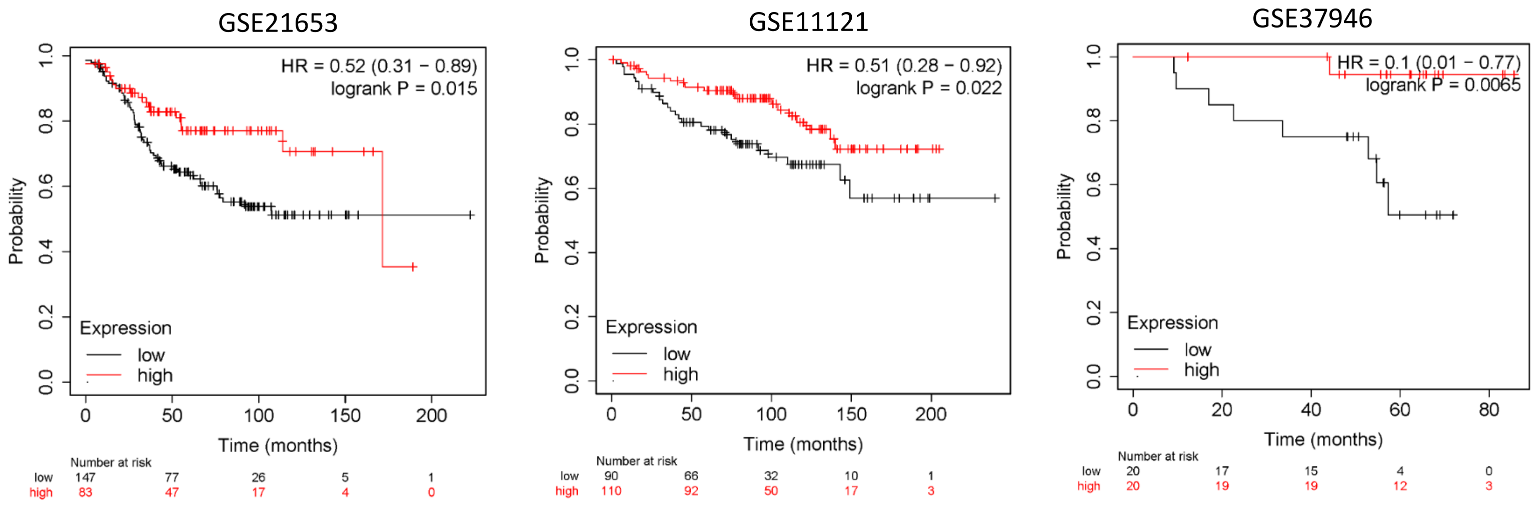

F

Study ID	Breast cancer Characteristics	Treatment
GSE17705	ER-positive	Tamoxifen
GSE9195	ER-positive	Tamoxifen
GSE48390	ER-positive or HER2-positive	No neoadjuvant chemotherapy
GSE26971	Early stage ER-positive	Tamoxifen
GSE7390	Lymph node negative early stage ER-positive and ER-negative	With and without systemic therapy
GSE21653	Medullary breast cancers	Unknown
GSE11121	Lymph node negative hormone receptor-positive and hormone receptor-negative	No systemic therapy
GSE37946	HER2+:ERα- breast cancer	Trastuzumab-based chemotherapy

Figure S1. TMEM176B expression in human breast cancer samples.

(A) TMEM176B gene copy number alterations in the METABRIC dataset by PAM50 subtype. (B) *TMEM176B* mRNA expression in different breast cancer subclasses. Plot was generated by UALCAN Portal with data from TCGA. TNBC: Triple negative breast cancer; BL1: basal-like 1; BL-2: basal-like 2; IM: immunomodulatory; M: mesenchymal; MSL: mesenchymal stem cell-like; LAR: luminal androgen receptor; UNS: unspecified. Normal vs TNBC-IM $p=3.73 \times 10^{-5}$, Luminal vs TNBC-IM $p = 8.41 \times 10^{-5}$ (C) *TMEM176B* gene expression in different breast cancer grades. Plot was generated by GENT2. Grade 1 vs Grade 2 p=0.024, Grade 1 vs Grade 3 p=0.005, Grade 2 vs Grade 3 p=0.664. (D-E) Relapse-free survival (RFS) plots according to TMEM176B expression levels. Plots were generated by Kaplan-Meier plotter. (F) Summary of the survival studies.

Figure S2.

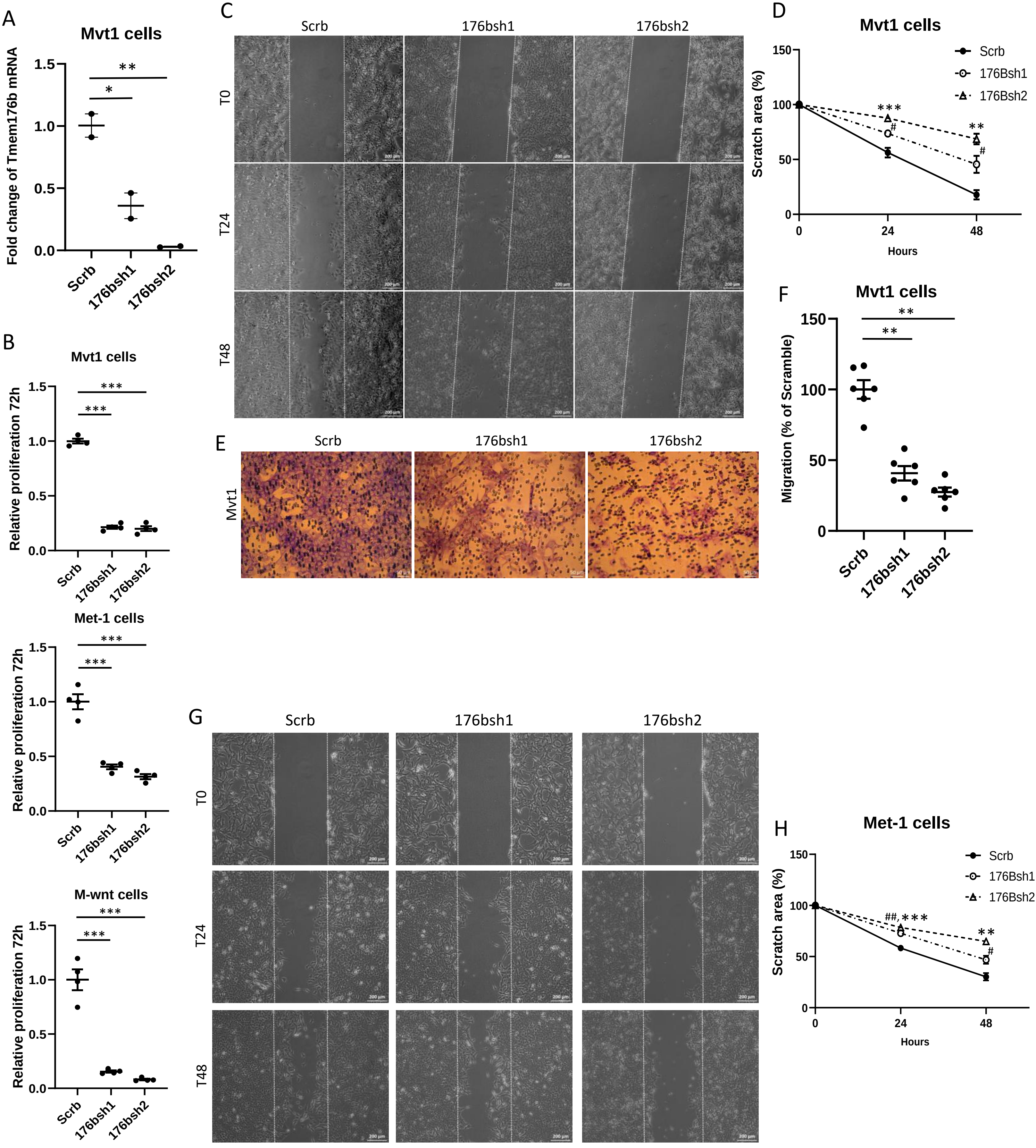
A
Mvt1 cells
Fold change of Tmem176b mRNA
Scrb
176bsh1
176bsh2
B
Mvt1 cells
Met-1 cells
M-wnt cells
Relative proliferation 72h
C
Scrb
176bsh1
176bsh2
T0
T24
T48
D
Mvt1 cells
Scratch area (%)
Hours
176Bsh1
176Bsh2
E
Mvt1
F
Mvt1 cells
Migration (% of Scramble)
G
H
Met-1 cells

Figure S2.

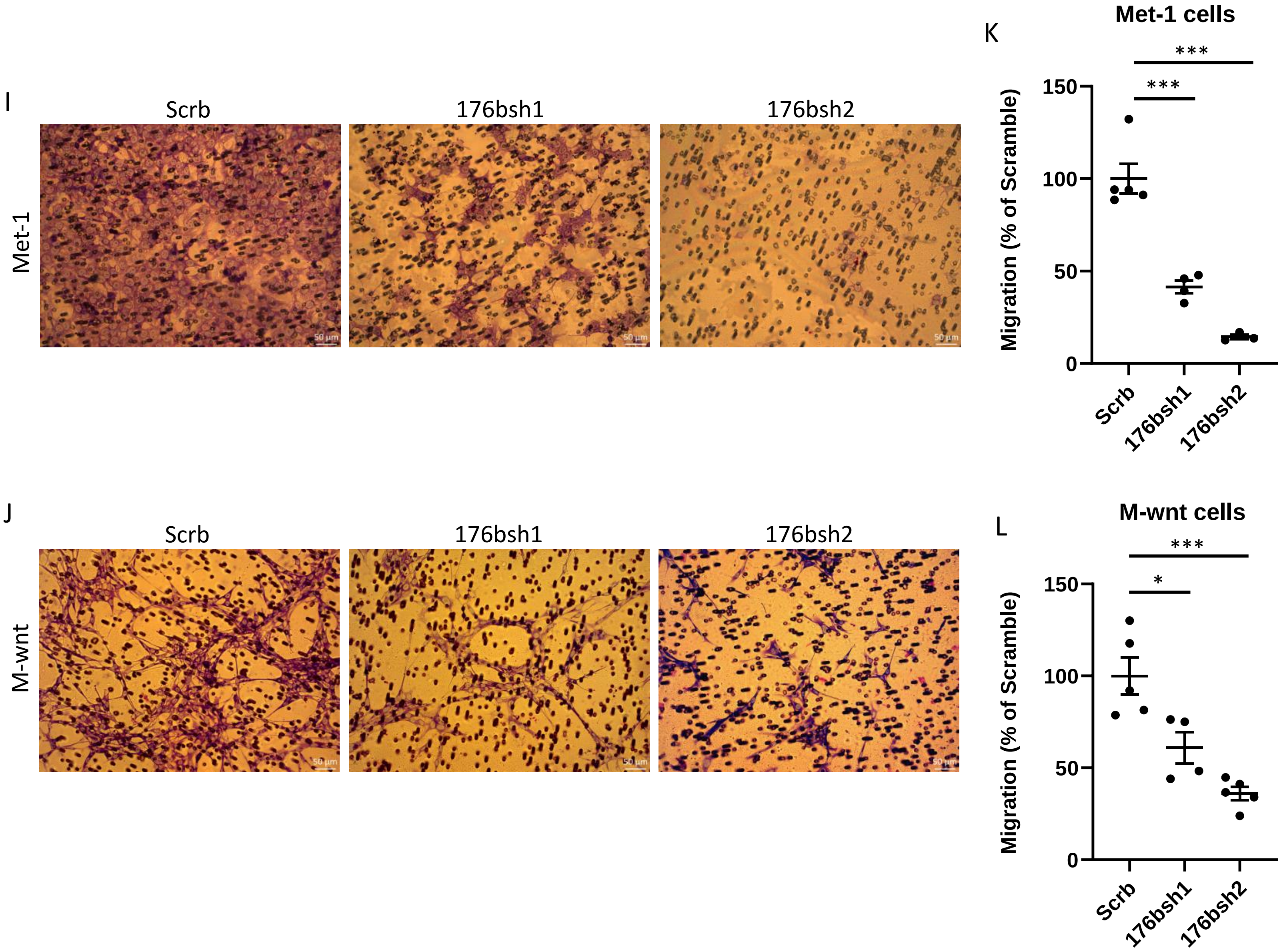
I
Scrb
176bsh1
176bsh2
Met-1
K
Met-1 cells
***
***
Migration (% of Scramble)
150
100
50
0
Scrb
176bsh1
176bsh2
J
Scrb
176bsh1
176bsh2
M-wnt
L
M-wnt cells
***
*
Migration (% of Scramble)
150
100
50
0
Scrb
176bsh1
176bsh2

Figure S2. Tmem176b knockdown decreases the proliferation rate and migration of murine mammary tumor cells in vitro.

(A) Control (Scrb) and two different shRNA constructs shRNA#1 (176bsh1) and #2 (176bsh2), were used to generate cell lines with *Tmem176b* silenced, as indicated. *Tmem176b* mRNA expression was assessed by qRT-PCR (n=2 per group in one independent experiment). (B) Proliferation assay for control (Scrb) and Tmem176b silenced Mvt1, Met-1 and M-wnt cells after 72 hours (n=4 per group, with two independent experiments). The proliferation rate was measured relative to the control cells and calculated as the ratio between the fluorescent values at the end of the experiment and time 0. (C) Wound healing assay for control and Tmem176b silenced Mvt1 cells. Images were taken at time points 0, 24, and 48 hours after performing the scratch at the same coordinates for each image. Scale bars: 200 μm. Images are representative from two independent experiments. (D) Quantification of wound healing assay expressed as the percent of scratch area remaining relative to time 0 (n=3 per group, with two independent experiments). (E) Representative images of transwell assays of Mvt1 cells after 20 hours of migration and after staining with Giemsa solution. Scale bars, 50 μm. (F) Quantification of transwell migration assays presented as percent of area stained relative to of control cells (n=6 per group, with three independent experiments). (G) Wound healing assay for Met-1 cells. (H) Quantification of wound healing assay in Met-1 cells (n=3 per group, with two independent experiments). (I) Met-1 cell and (J) M-wnt cell transwell migration assay. Quantification of transwell migration assay for (K) Met-1 cell (n=5 for Scrb; n=4 for 176bsh1 and n=3 for 176bsh2 per group, with three independent experiments) and (L) M-wnt cell (n=5 for Scrb; n=4 for 176bsh1 and n=5 for 176bsh2 per group, with three independent experiments). Data are presented as mean ± SEM. Differences between groups were evaluated by one-way (A, B, D, F,

H, J and L) ANOVA test with Bonferroni post hoc test. *or #P < 0.05, **or ##P < 0.01, ***or ###P < 0.001. (Scrb vs. 176bsh1 #; Scrb vs. 176bsh2 *)

Figure S3.

A

TCGA Dataset – TGCA Provisional - cbioportal

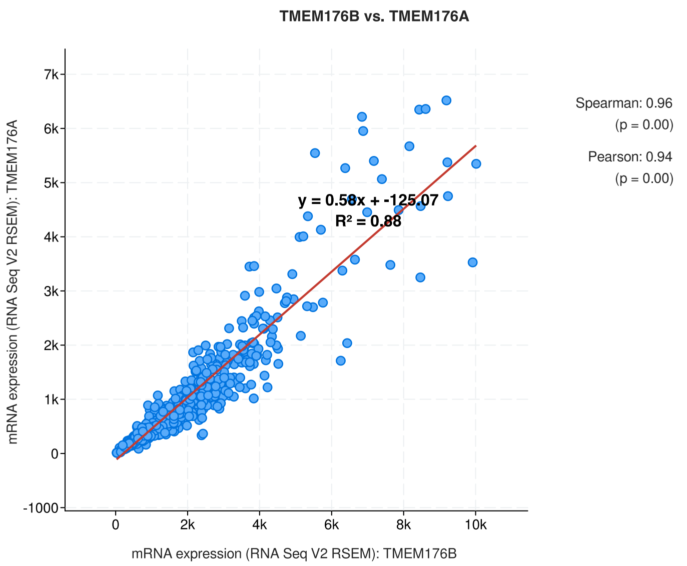

B

METABRIC – BREAST CANCER - cbioportal

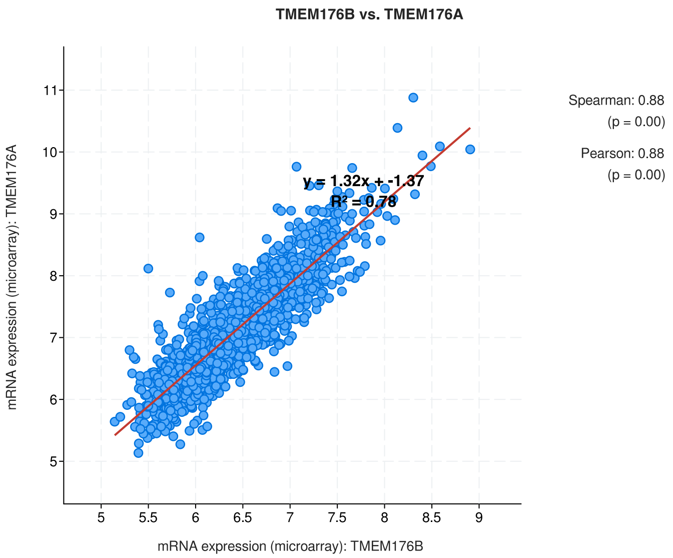

Figure S3. Correlation of *TMEM176A* and *TMEM176B*.

Correlation of mRNA expression of *TMEM176A* and *TMEM176B* in (A) TCGA and (B) METABRIC human breast cancer datasets generated using cBioPortal for Cancer Genomics.

Figure S4.

A

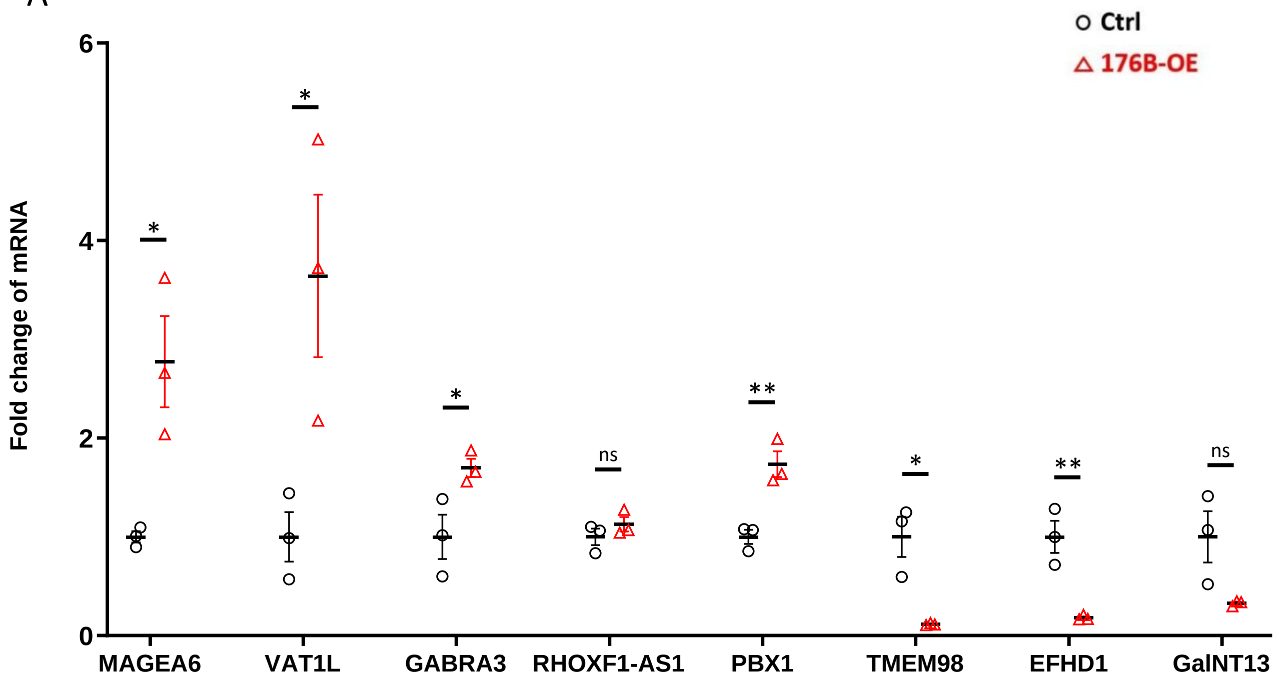
Ctrl
176B-OE
Fold change of mRNA
6
4
2
0
*
*
*
ns
**
*
**
ns
MAGEA6
VAT1L
GABRA3
RHOXF1-AS1
PBX1
TMEM98
EFHD1
GalNT13

B

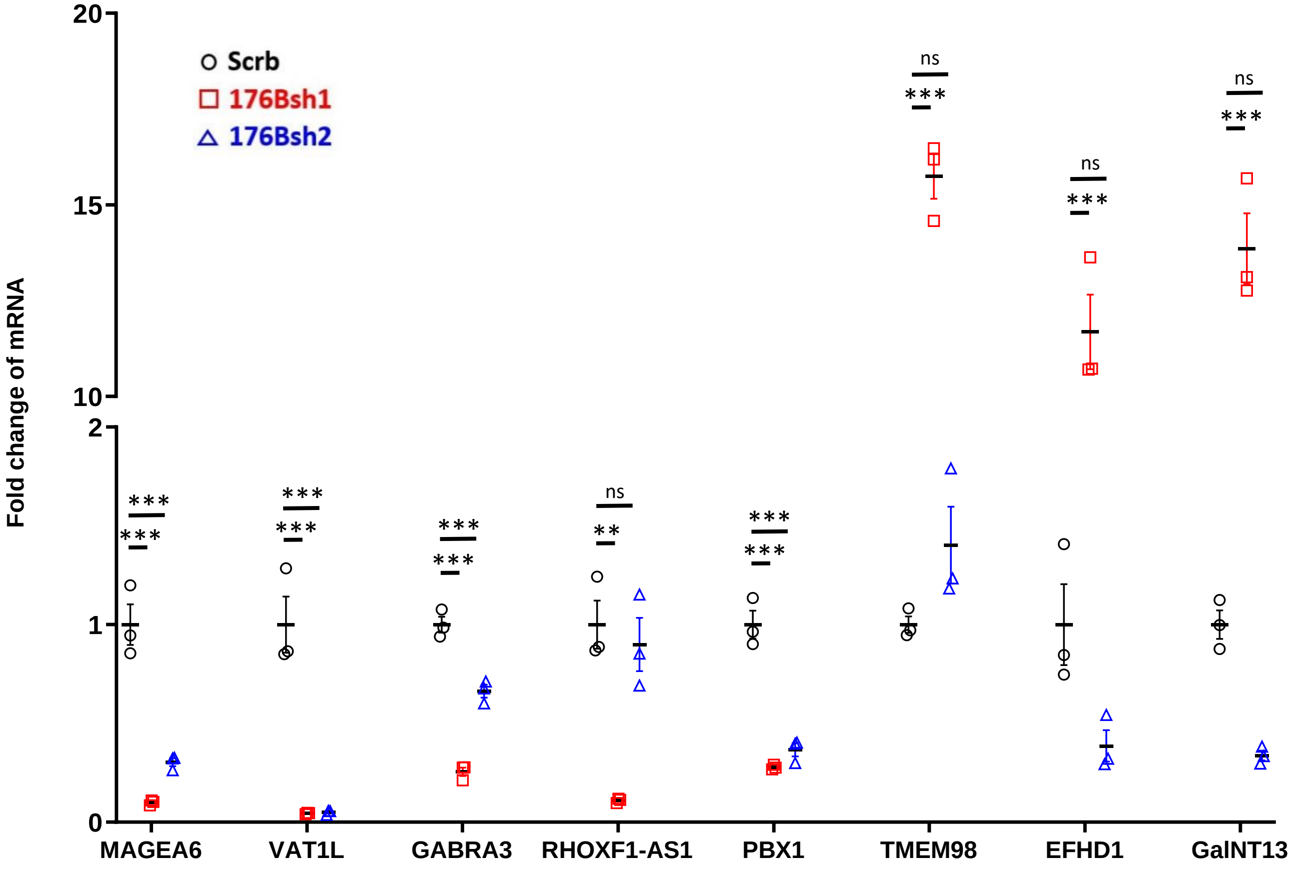
Scrb
176Bsh1
176Bsh2
Fold change of mRNA
20
15
10
2
1
0
ns
***
ns
***
ns
***
***
***
***
***
***
***
ns
**
***
***
MAGEA6
VAT1L
GABRA3
RHOXF1-AS1
PBX1
TMEM98
EFHD1
GalNT13

Figure S4. Validation of RNA-Sequencing results by qRT-PCR.

Differentially expressed genes in RNA-Seq were validated using qRT-qPCR. Ct values were normalized to housekeeping gene RPL19. Data are presented as means ± SEM. (A) Differences between MDA-MB-231 control and TMEM176B overexpressing cell groups were evaluated by student’s t-test. (B) Differences between MDA-MB-231 control (Scrb) and TMEM176B silenced cell groups were by one-way ANOVA test with Bonferroni post hoc test. ns: no significant; *P < 0.05, **P < 0.01, ***P < 0.001.

Figure S5.

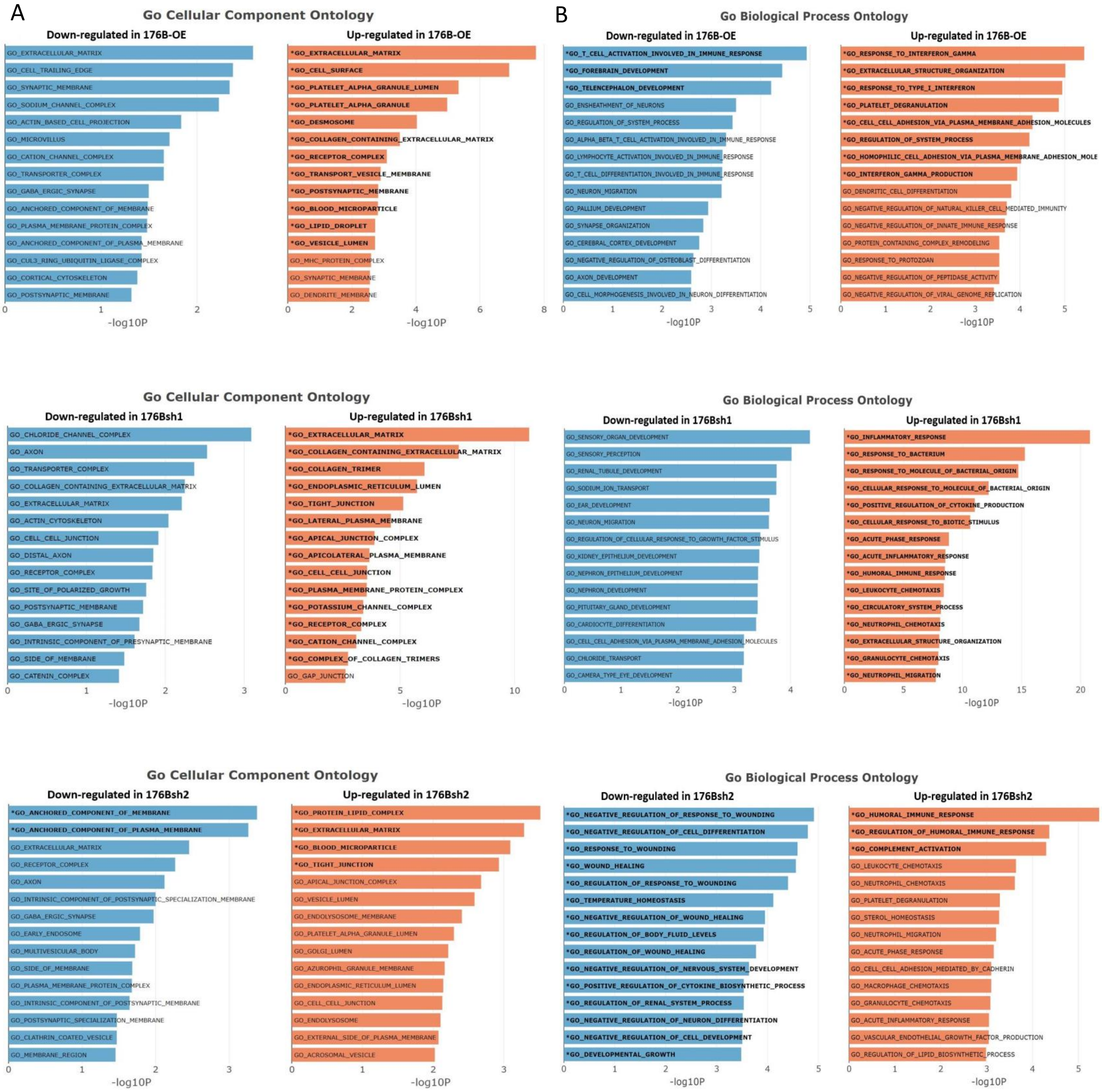
A
Go Cellular Component Ontology
Down-regulated in 176B-OE
GO_EXTRACELLULAR_MATRIX
GO_CELL_TRAILING_EDGE
GO_SYNAPTIC_MEMBRANE
GO_SODIUM_CHANNEL_COMPLEX
GO_ACTIN_BASED_CELL_PROJECTION
GO_MICROVILLUS
GO_CATION_CHANNEL_COMPLEX
GO_TRANSPORTER_COMPLEX
GO_GABA_ERGIC_SYNAPSE
GO_ANCHORED_COMPONENT_OF_MEMBRANE
GO_PLASMA_MEMBRANE_PROTEIN_COMPLEX
GO_ANCHORED_COMPONENT_OF_PLASMA_MEMBRANE
GO_CUL3_RING_UBIQUITIN_LIGASE_COMPLEX
GO_CORTICAL_CYTOSKELETON
GO_POSTSYNAPTIC_MEMBRANE
-log10P
Up-regulated in 176B-OE
*GO_EXTRACELLULAR_MATRIX
*GO_CELL_SURFACE
*GO_PLATELET_ALPHA_GRANULE_LUMEN
*GO_PLATELET_ALPHA_GRANULE
*GO_DESMOSOME
*GO_COLLAGEN_CONTAINING_EXTRACELLULAR_MATRIX
*GO_RECEPTOR_COMPLEX
*GO_TRANSPORT_VESICLE_MEMBRANE
*GO_POSTSYNAPTIC_MEMBRANE
*GO_BLOOD_MICROPARTICLE
*GO_LIPID_DROPLET
*GO_VESICLE_LUMEN
GO_MHC_PROTEIN_COMPLEX
GO_SYNAPTIC_MEMBRANE
GO_DENDRITE_MEMBRANE
-log10P
B
Go Biological Process Ontology
Down-regulated in 176B-OE
*GO_T_CELL_ACTIVATION_INVOLVED_IN_IMMUNE_RESPONSE
*GO_FOREBRAIN_DEVELOPMENT
*GO_TELENCEPHALON_DEVELOPMENT
GO_ENSHEATHMENT_OF_NEURONS
GO_REGULATION_OF_SYSTEM_PROCESS
GO_ALPHA_BETA_T_CELL_ACTIVATION_INVOLVED_IN_IMMUNE_RESPONSE
GO_LYMPHOCYTE_ACTIVATION_INVOLVED_IN_IMMUNE_RESPONSE
GO_T_CELL_DIFFERENTIATION_INVOLVED_IN_IMMUNE_RESPONSE
GO_NEURON_MIGRATION
GO_PALLIUM_DEVELOPMENT
GO_SYNAPSE_ORGANIZATION
GO_CEREBRAL_CORTEX_DEVELOPMENT
GO_NEGATIVE_REGULATION_OF_OSTEOBLAST_DIFFERENTIATION
GO_AXON_DEVELOPMENT
GO_CELL_MORPHOGENESIS_INVOLVED_IN_NEURON_DIFFERENTIATION
-log10P
Up-regulated in 176B-OE
*GO_RESPONSE_TO_INTERFERON_GAMMA
*GO_EXTRACELLULAR_STRUCTURE_ORGANIZATION
*GO_RESPONSE_TO_TYPE_I_INTERFERON
*GO_PLATELET_DEGRANULATION
*GO_CELL_CELL_ADHESION_VIA_PLASMA_MEMBRANE_ADHESION_MOLECULES
*GO_REGULATION_OF_SYSTEM_PROCESS
*GO_HOMOPHILIC_CELL_ADHESION_VIA_PLASMA_MEMBRANE_ADHESION_MOLE
*GO_INTERFERON_GAMMA_PRODUCTION
GO_DENDRITIC_CELL_DIFFERENTIATION
GO_NEGATIVE_REGULATION_OF_NATURAL_KILLER_CELL_MEDIATED_IMMUNITY
GO_NEGATIVE_REGULATION_OF_INNATE_IMMUNE_RESPONSE
GO_PROTEIN_CONTAINING_COMPLEX_REMODELING
GO_RESPONSE_TO_PROTOZOAN
GO_NEGATIVE_REGULATION_OF_PEPTIDASE_ACTIVITY
GO_NEGATIVE_REGULATION_OF_VIRAL_GENOME_REPLICATION
-log10P
Go Cellular Component Ontology
Down-regulated in 176Bsh1
GO_CHLORIDE_CHANNEL_COMPLEX
GO_AXON
GO_TRANSPORTER_COMPLEX
GO_COLLAGEN_CONTAINING_EXTRACELLULAR_MATRIX
GO_EXTRACELLULAR_MATRIX
GO_ACTIN_CYTOSKELETON
GO_CELL_CELL_JUNCTION
GO_DISTAL_AXON
GO_RECEPTOR_COMPLEX
GO_SITE_OF_POLARIZED_GROWTH
GO_POSTSYNAPTIC_MEMBRANE
GO_GABA_ERGIC_SYNAPSE
GO_INTRINSIC_COMPONENT_OF_PRESYNAPTIC_MEMBRANE
GO_SIDE_OF_MEMBRANE
GO_CATENIN_COMPLEX
-log10P
Up-regulated in 176Bsh1
*GO_EXTRACELLULAR_MATRIX
*GO_COLLAGEN_CONTAINING_EXTRACELLULAR_MATRIX
*GO_COLLAGEN_TRIMER
*GO_ENDOPLASMIC_RETICULUM_LUMEN
*GO_TIGHT_JUNCTION
*GO_LATERAL_PLASMA_MEMBRANE
*GO_APICAL_JUNCTION_COMPLEX
*GO_APICOLATERAL_PLASMA_MEMBRANE
*GO_CELL_CELL_JUNCTION
*GO_PLASMA_MEMBRANE_PROTEIN_COMPLEX
*GO_POTASSIUM_CHANNEL_COMPLEX
*GO_RECEPTOR_COMPLEX
*GO_CATION_CHANNEL_COMPLEX
*GO_COMPLEX_OF_COLLAGEN_TRIMERS
GO_GAP_JUNCTION
-log10P
Go Biological Process Ontology
Down-regulated in 176Bsh1
GO_SENSORY_ORGAN_DEVELOPMENT
GO_SENSORY_PERCEPTION
GO_RENAL_TUBULE_DEVELOPMENT
GO_SODIUM_ION_TRANSPORT
GO_EAR_DEVELOPMENT
GO_NEURON_MIGRATION
GO_REGULATION_OF_CELLULAR_RESPONSE_TO_GROWTH_FACTOR_STIMULUS
GO_KIDNEY_EPITHELIUM_DEVELOPMENT
GO_NEPHRON_EPITHELIUM_DEVELOPMENT
GO_NEPHRON_DEVELOPMENT
GO_PITUITARY_GLAND_DEVELOPMENT
GO_CARDIOCYTE_DIFFERENTIATION
GO_CELL_CELL_ADHESION_VIA_PLASMA_MEMBRANE_ADHESION_MOLECULES
GO_CHLORIDE_TRANSPORT
GO_CAMERA_TYPE_EYE_DEVELOPMENT
-log10P
Up-regulated in 176Bsh1
*GO_INFLAMMATORY_RESPONSE
*GO_RESPONSE_TO_BACTERIUM
*GO_RESPONSE_TO_MOLECULE_OF_BACTERIAL_ORIGIN
*GO_CELLULAR_RESPONSE_TO_MOLECULE_OF_BACTERIAL_ORIGIN
*GO_POSITIVE_REGULATION_OF_CYTOKINE_PRODUCTION
*GO_CELLULAR_RESPONSE_TO_BIOTIC_STIMULUS
*GO_ACUTE_PHASE_RESPONSE
*GO_ACUTE_INFLAMMATORY_RESPONSE
*GO_HUMORAL_IMMUNE_RESPONSE
*GO_LEUKOCYTE_CHEMOTAXIS
*GO_CIRCULATORY_SYSTEM_PROCESS
*GO_NEUTROPHIL_CHEMOTAXIS
*GO_EXTRACELLULAR_STRUCTURE_ORGANIZATION
*GO_GRANULOCYTE_CHEMOTAXIS
*GO_NEUTROPHIL_MIGRATION
-log10P
Go Cellular Component Ontology
Down-regulated in 176Bsh2
*GO_ANCHORED_COMPONENT_OF_MEMBRANE
*GO_ANCHORED_COMPONENT_OF_PLASMA_MEMBRANE
GO_EXTRACELLULAR_MATRIX
GO_RECEPTOR_COMPLEX
GO_AXON
GO_INTRINSIC_COMPONENT_OF_POSTSYNAPTIC_SPECIALIZATION_MEMBRANE
GO_GABA_ERGIC_SYNAPSE
GO_EARLY_ENDOSOME
GO_MULTIVESICULAR_BODY
GO_SIDE_OF_MEMBRANE
GO_PLASMA_MEMBRANE_PROTEIN_COMPLEX
GO_INTRINSIC_COMPONENT_OF_POSTSYNAPTIC_MEMBRANE
GO_POSTSYNAPTIC_SPECIALIZATION_MEMBRANE
GO_CLATHRIN_COATED_VESICLE
GO_MEMBRANE_REGION
-log10P
Up-regulated in 176Bsh2
*GO_PROTEIN_LIPID_COMPLEX
*GO_EXTRACELLULAR_MATRIX
*GO_BLOOD_MICROPARTICLE
*GO_TIGHT_JUNCTION
GO_APICAL_JUNCTION_COMPLEX
GO_VESICLE_LUMEN
GO_ENDOLYSOSOME_MEMBRANE
GO_PLATELET_ALPHA_GRANULE_LUMEN
GO_GOLGI_LUMEN
GO_AZUROPHIL_GRANULE_MEMBRANE
GO_ENDOPLASMIC_RETICULUM_LUMEN
GO_CELL_CELL_JUNCTION
GO_ENDOLYSOSOME
GO_EXTERNAL_SIDE_OF_PLASMA_MEMBRANE
GO_ACROSOMAL_VESICLE
-log10P
Go Biological Process Ontology
Down-regulated in 176Bsh2
*GO_NEGATIVE_REGULATION_OF_RESPONSE_TO_WOUNDING
*GO_NEGATIVE_REGULATION_OF_CELL_DIFFERENTIATION
*GO_RESPONSE_TO_WOUNDING
*GO_WOUND_HEALING
*GO_REGULATION_OF_RESPONSE_TO_WOUNDING
*GO_TEMPERATURE_HOMEOSTASIS
*GO_NEGATIVE_REGULATION_OF_WOUND_HEALING
*GO_REGULATION_OF_BODY_FLUID_LEVELS
*GO_REGULATION_OF_WOUND_HEALING
*GO_NEGATIVE_REGULATION_OF_NERVOUS_SYSTEM_DEVELOPMENT
*GO_POSITIVE_REGULATION_OF_CYTOKINE_BIOSYNTHETIC_PROCESS
*GO_REGULATION_OF_RENAL_SYSTEM_PROCESS
*GO_NEGATIVE_REGULATION_OF_NEURON_DIFFERENTIATION
*GO_NEGATIVE_REGULATION_OF_CELL_DEVELOPMENT
*GO_DEVELOPMENTAL_GROWTH
-log10P
Up-regulated in 176Bsh2
*GO_HUMORAL_IMMUNE_RESPONSE
*GO_REGULATION_OF_HUMORAL_IMMUNE_RESPONSE
*GO_COMPLEMENT_ACTIVATION
GO_LEUKOCYTE_CHEMOTAXIS
GO_NEUTROPHIL_CHEMOTAXIS
GO_PLATELET_DEGRANULATION
GO_STEROL_HOMEOSTASIS
GO_NEUTROPHIL_MIGRATION
GO_ACUTE_PHASE_RESPONSE
GO_CELL_CELL_ADHESION_MEDIATED_BY_CADHERIN
GO_MACROPHAGE_CHEMOTAXIS
GO_GRANULOCYTE_CHEMOTAXIS
GO_ACUTE_INFLAMMATORY_RESPONSE
GO_VASCULAR_ENDOTHELIAL_GROWTH_FACTOR_PRODUCTION
GO_REGULATION_OF_LIPID_BIOSYNTHETIC_PROCESS
-log10P

Figure S5. Functional analysis of Gene Ontology gene sets from RNA-Seq data.

Comparison of gene expression differences between control and TMEM176B overexpressing cells as well as shRNA control (Scrb) and TMEM176B silenced (176Bsh1, 176Bsh2) cells in (A) Go cellular component ontology and (B) Go biological process ontology. Gene sets reported in bold with an asterisk are significantly enriched at adjusted p-value < 0.05.

Table S1. Plasmid list

Vectors description	Catalog number	company
control construct for mouse *Tmem176b* knockdown	CSHCTR001-LVRU6GP	Genecopoeia (Rockville, MD, USA)
mouse *Tmem176b* knockdown construct shRNA-1 (176bsh1)	MSH032900-5-LVRU6GP	Genecopoeia (Rockville, MD, USA)
mouse *Tmem176b* knockdown construct shRNA-1 (176bsh2)	MSH032900-8-LVRU6GP	Genecopoeia (Rockville, MD, USA)
control construct for human *TMEM176B* knockdown	CSHCTR001-LVRU6GP	Genecopoeia (Rockville, MD, USA)
human *TMEM176B* knockdown construct shRNA-1 (176Bsh1)	HSH0083210-22-LVRU6GP	Genecopoeia (Rockville, MD, USA)
human *TMEM176B* knockdown construct shRNA-1 (176Bsh1)	HSH0083210-23-LVRU6GP	Genecopoeia (Rockville, MD, USA)
control construct for human *TMEM176A* knockdown	CSHCTR001-LVRU6MP	Genecopoeia (Rockville, MD, USA)
human *TMEM176A* knockdown construct shRNA-1 (176Ash1)	HSH014372-31-LVRU6MP	Genecopoeia (Rockville, MD, USA)
human *TMEM176A* knockdown construct shRNA-1 (176Ash2)	HSH014372-32-LVRU6MP	Genecopoeia (Rockville, MD, USA)
control construct for human TMEM176A and TMEM176B encoding constructs	PS100001	OriGene (Rockville, MD, USA)
human TMEM176A encoding construct (176A-OE)	RC203433	OriGene (Rockville, MD, USA)
human TMEM176B encoding construct (176B-OE)	RC200802	OriGene (Rockville, MD, USA)
Pax2	35002	Addgene (Cambridge, MA, USA)
pCMV-VSV-G	8454	Addgene (Cambridge, MA, USA)

Table S2. In vivo studies detailed information

<table>
<tr><th></th><th>Animal strain</th><th>Cell lines</th><th colspan="2">In each group n=</th><th>Total animals used</th></tr>
<tr><td rowspan="3">Study 1</td><td rowspan="3">FVB/N background WT female mice</td><td rowspan="3">Scrambled and Tmem176b knockdown Mvt1 cells</td><td>Scrb</td><td>5*</td><td rowspan="3">20</td></tr>
<tr><td>176bsh1</td><td>6**</td></tr>
<tr><td>176bsh2</td><td>7</td></tr>
<tr><td rowspan="3">Study 2</td><td rowspan="3">FVB/N background Rag1$^{-/-}$ female mice</td><td rowspan="3">Scrambled and TMEM176B knockdown MDA-MB-231 cells</td><td>Scrb</td><td>6</td><td rowspan="3">18</td></tr>
<tr><td>176Bsh1</td><td>6</td></tr>
<tr><td>176Bsh2</td><td>6</td></tr>
<tr><td rowspan="3">Study 3</td><td rowspan="3">FVB/N background Rag1$^{-/-}$ female mice</td><td rowspan="3">Scrambled and TMEM176A knockdown MDA-MB-231 cells</td><td>Scrb</td><td>7***</td><td rowspan="3">12</td></tr>
<tr><td>176Ash1</td><td>8</td></tr>
<tr><td>176Ash2</td><td>8</td></tr>
</table>

*This group originally numbered 6 animals but one mouse tumor size is an outlier. It was removed from the study.

**This group originally numbered 7 animals but one mouse tumor size record was incomplete. It was removed from the study.

***This group originally numbered 8 animals but one mouse tumor regressed. It was removed from the study.

Table S3. Primer list

Primers description	Sequence	Catalog number	Company
Mouse *Tmem176b* primer Forward	CATCAGCATCCACATCCACC	N/A	Eurofins Genomics LLC (Louisville, KY, USA)
Mouse *Tmem176b* primer Reverse	ACTCCAGCTAGAATTGCCACAG	N/A	Eurofins Genomics LLC (Louisville, KY, USA)
Mouse *B2M* primer Forward	TTCTGGTGCTTGTCTCACTGA	N/A	Eurofins Genomics LLC (Louisville, KY, USA)
Mouse *B2M* primer Reverse	CAGTATGTTCGGCTTCCCATTC	N/A	Eurofins Genomics LLC (Louisville, KY, USA)
Human *RPL19* primer Forward	AGCTCTTTCCTTTCGCTGCT	N/A	Eurofins Genomics LLC (Louisville, KY, USA)
Human *RPL19* primer Reverse	GATCTGCTGACGGGAGTTGG	N/A	Eurofins Genomics LLC (Louisville, KY, USA)
Human *TMEM176A* primes pair	N/A	HQP014372	Genecopoeia (Rockville, MD, USA)
Human *TMEM176B* primes pair	N/A	HQP008320	Genecopoeia (Rockville, MD, USA)

Table S4. Antibody list

Antibodies	dilution	Catalog number	Company
TMEM176B	~400µg/ml	customized	ProSci (Poway, CA, USA)
TMEM176A			Gift from Dr. Math P. Cuajungco
Akt	1:1000	#9272	Cell Signaling Technology (Danvers, MA, USA)
Phospho-Akt (Thr^{308})	1:1000	#13038	Cell Signaling Technology (Danvers, MA, USA)
Phospho-Akt (Ser^{473})	1:1000	#9271	Cell Signaling Technology (Danvers, MA, USA)
RSK1/RSK2/RSK3	1:1000	#9355	Cell Signaling Technology (Danvers, MA, USA)
Phospho-p90RSK (Ser^{380})	1:1000	#11989	Cell Signaling Technology (Danvers, MA, USA)
p70 S6 Kinase	1:1000	#9202	Cell Signaling Technology (Danvers, MA, USA)
Phospho-p70 S6 Kinase (Thr^{389})	1:1000	#9234	Cell Signaling Technology (Danvers, MA, USA)
Total S6 Ribosomal Protein	1:1000	#2317	Cell Signaling Technology (Danvers, MA, USA)
Phospho-S6 Ribosomal Protein ($Ser^{235/236}$)	1:1000	#2211	Cell Signaling Technology (Danvers, MA, USA)
β-Actin	1:5000	A5441	Sigma-Aldrich (St. Louis, MO, USA)
IRDye® 680RD Goat anti-Mouse IgG	1:5000	926-68070	LI-COR Biosciences (Lincoln, NE, USA)
IRDye® 800CW Goat anti-Rabbit IgG	1:5000	926-32211	LI-COR Biosciences (Lincoln, NE, USA)
TMEM176B	1:100	HPA047509	Sigma-Aldrich (St. Louis, MO, USA)
Goat anti-Rabbit IgG (H+L) Secondary Antibody, Alexa Fluor 568	1:200	A-21069	Thermo Fisher Scientific (Waltham, MA, USA)
Alexa Fluor™ 647 Phalloidin	1:400	A22287	Thermo Fisher Scientific (Waltham, MA, USA)
ProLong® Gold Antifade Reagent with DAPI		#8961	Cell Signaling Technology (Danvers, MA, USA)

Table S5. RNA-Seq validation primers list

Primers description	Sequence	Company
MAGEA6 primer Forward	CTTCCTGATAATCATCCTGGCC	Eurofins Genomics LLC (Louisville, KY, USA)
MAGEA6 primer Reverse	GAAATATTGGGTGAGCAGCTTC	Eurofins Genomics LLC (Louisville, KY, USA)
VAT1L primer Forward	GACTGTCTTTGGAACAGCCTCTA	Eurofins Genomics LLC (Louisville, KY, USA)
VAT1L primer Reverse	ATGTCCACACCTTCAGCAGAG	Eurofins Genomics LLC (Louisville, KY, USA)
GABRA3 primer Forward	AATCTTGGATCGTCTTCTGGAC	Eurofins Genomics LLC (Louisville, KY, USA)
GABRA3 primer Reverse	AAACTGGTCACGTAGATGTCAG	Eurofins Genomics LLC (Louisville, KY, USA)
RHOXF1-AS1 primer Forward	GATCCACCTGGTGTGATGAAT	Eurofins Genomics LLC (Louisville, KY, USA)
RHOXF1-AS1 primer Reverse	AGAGTTTCCTGGTTCACGTGG	Eurofins Genomics LLC (Louisville, KY, USA)
PBX1 primer Forward	TTGGAAATAAGCGAATCCGGTA	Eurofins Genomics LLC (Louisville, KY, USA)
PBX1 primer Reverse	TGACACATTGGTAGCAGTGACA	Eurofins Genomics LLC (Louisville, KY, USA)
TMEM98 primer Forward	CGCTATGATTCTAAGCCCATTG	Eurofins Genomics LLC (Louisville, KY, USA)
TMEM98 primer Reverse	GTTGGTGATAACGACATCGTCC	Eurofins Genomics LLC (Louisville, KY, USA)
EFHD1 primer Forward	GTTGAAAGCTGAGCAAGATGAG	Eurofins Genomics LLC (Louisville, KY, USA)
EFHD1 primer Reverse	CTATGTATTGAAGTTGGCCTTGAG	Eurofins Genomics LLC (Louisville, KY, USA)
GALNT13 primer Forward	TAATACGTGCCCGTCTTCGAG	Eurofins Genomics LLC (Louisville, KY, USA)
GALNT13 primer Reverse	CTTGCCAGCAAAGGCTCCAG	Eurofins Genomics LLC (Louisville, KY, USA)
